# *Quo vadis* Cardiac Glycoside Research?

**DOI:** 10.3390/toxins13050344

**Published:** 2021-05-11

**Authors:** Jiří Bejček, Michal Jurášek, Vojtěch Spiwok, Silvie Rimpelová

**Affiliations:** 1 Department of Biochemistry and Microbiology, University of Chemistry and Technology Prague, Technická 5, Prague 6, Czech Republic; jiri.bejcek@vscht.cz (J.B.); vojtech.spiwok@vscht.cz (V.S.); 2 Department of Chemistry of Natural Compounds, University of Chemistry and Technology Prague, Technická 3, Prague 6, Czech Republic; michal.jurasek@vscht.cz

**Keywords:** cancer treatment, cardenolides, digitoxin, digoxin, drug repositioning, immunogenic cell death, Na^+^/K^+^ ATPase, antiviral potential, secondary plant metabolites, toxins

## Abstract

Cardiac glycosides (CGs), toxins well-known for numerous human and cattle poisoning, are natural compounds, the biosynthesis of which occurs in various plants and animals as a self-protective mechanism to prevent grazing and predation. Interestingly, some insect species can take advantage of the CG’s toxicity and by absorbing them, they are also protected from predation. The mechanism of action of CG’s toxicity is inhibition of Na^+^/K^+^-ATPase (the sodium-potassium pump, NKA), which disrupts the ionic homeostasis leading to elevated Ca^2+^ concentration resulting in cell death. Thus, NKA serves as a molecular target for CGs (although it is not the only one) and even though CGs are toxic for humans and some animals, they can also be used as remedies for various diseases, such as cardiovascular ones, and possibly cancer. Although the anticancer mechanism of CGs has not been fully elucidated, yet, it is thought to be connected with the second role of NKA being a receptor that can induce several cell signaling cascades and even serve as a growth factor and, thus, inhibit cancer cell proliferation at low nontoxic concentrations. These growth inhibitory effects are often observed only in cancer cells, thereby, offering a possibility for CGs to be repositioned for cancer treatment serving not only as chemotherapeutic agents but also as immunogenic cell death triggers. Therefore, here, we report on CG’s chemical structures, production optimization, and biological activity with possible use in cancer therapy, as well as, discuss their antiviral potential which was discovered quite recently. Special attention has been devoted to digitoxin, digoxin, and ouabain.

## 1. Introduction

Biologically active secondary metabolites occur in almost any living system, from unicellular organisms to fungi, plants, and animals. Each organism producing these secondary metabolites has adapted its metabolism to its environment, and this adaptation enabled it to cope with the surrounding threats. Since life occurs in different environments with slightly different living conditions, the chemical nature of secondary metabolites differs as well. Most of the secondary metabolites, such as alkaloids [1], flavonoids [2,3], phytosterols [4], and many others, are found in the plants that “ran” the furthest in this imaginary race for survival. Although many secondary metabolites may, at the first sight, appear to be inherently toxic to humans and some other species, it is always important to keep in mind that at lower concentrations, these substances may have beneficial effects. It is for this reason that mankind presently uses these metabolites mainly in pharmacy.

**Figure 1:**
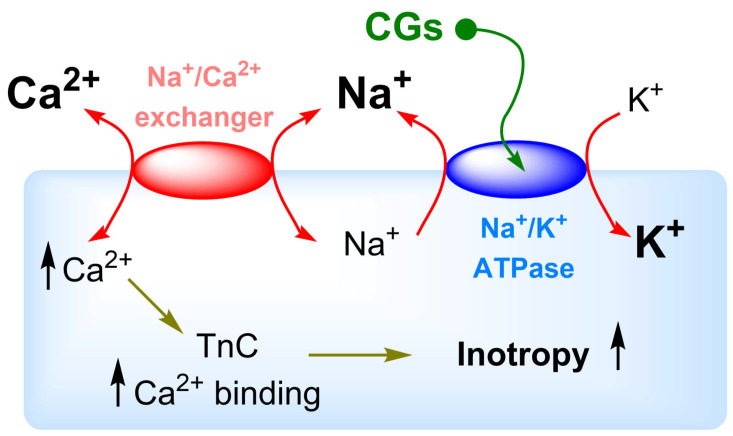
Cellular mechanism of cardiac glycoside action. TnC = troponin.

Among the most widely used secondary metabolites belong cardiac glycosides (CGs) which are among the 200 most commonly prescribed drugs in the USA (the year 2018) [5]. CGs are produced by several plants mainly as protection against pests [6,7]. Probably the best known is the production by plants of the *Digitalis* genus (Scrophulariaceae), from which the most important representatives of this group are mostly isolated, namely digoxin (**Dg**) and digitoxin (**Dgt**). Both substances are well-established drugs for the treatment of heart failure and cardiac arrhythmias, even though they have a relatively narrow therapeutic window that limits their use [8]. The mechanism of action of not only these representatives but also of the whole CG group is the inhibition of Na^+^/K^+^-ATPase (NKA), which causes a disruption of the ionic balance of the cell and, thus, leads to an increase in muscle contraction (Figure 1). However, Ca^2+^ ions, which serve as mediators of muscle contraction, are also involved in signaling pathways that ultimately lead to cell death by apoptosis. The latter fact is the reason why CGs are currently being studied as possible drugs for cancer treatment. Moreover, in recent decades (ca. 30 years), the fact that CGs can activate several signaling cascades involved in cell proliferation through interaction with NKA has come to light. It was revealed that CGs, via the interaction with NKA, can activate several signaling cascades that are involved in the regulation of cell proliferation, thereby, contributing to anticancer activity. The role of CGs in the regulation of blood pressure and, more recently, their antiviral effects were also discovered.

## 2. Occurrence of Cardiac Glycosides

CGs became more widely known to the medical community due to W. Withering, who used extracts from dried foxglove leaves to treat edema [9]. At that time, due to imperfect purification techniques, these extracts contained also other substances occurring in foxglove leaves, such as the already mentioned flavonoids [10], therefore, it was not possible then to determine which substances were responsible for the pharmacological effect of the extracts.

**Figure 2:**
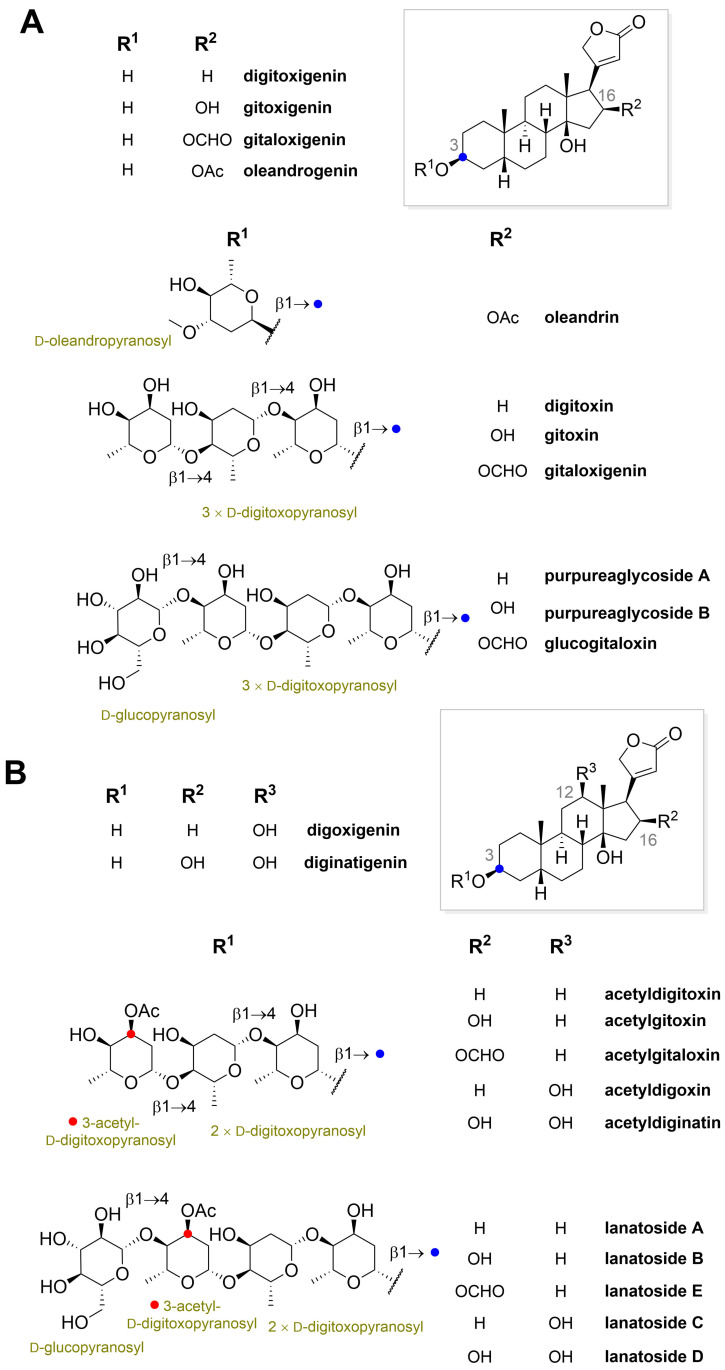
Chemical structures of C-16 (panel A symbol “
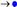
” highlight the configuration of linkage of sugar to C-3 hydroxyl of the steroid) and C-12 and C-16 (panel B) substituted cardiac glycosides (red dots highlight the acetylation on sugar moiety).

**Figure 3:**
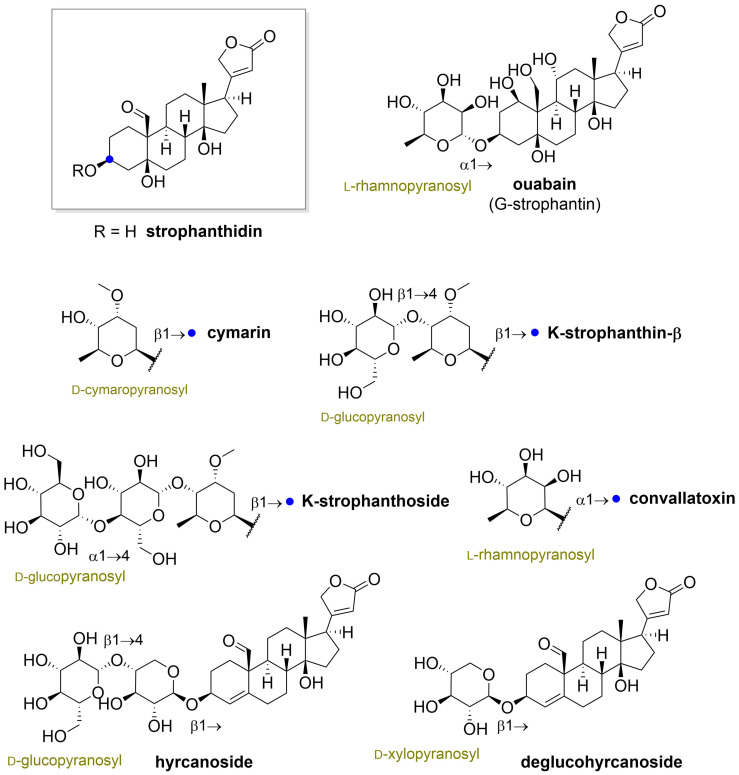
Chemical structures of natural C-19 formyl cardiac glycosides and ouabain (symbol “
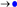
” highlight the configuration of linkage of sugar to C-3 hydroxyl of the steroid).

Nowadays, we know that the most important bioactive substances contained in these plants are CGs, and although many drugs are now produced by organic synthesis, CGs are still being isolated from foxglove (*Digitalis*), which are therefore their most important producers [11]. From Table 1, summarizing the most important producers of CGs in the plant kingdom, it is apparent that the plants always contain several structurally similar CGs. The most abundant CGs (Figure 2 and Figure 3) then usually have their name derived from the designation of the corresponding plant.

As is obvious from Table 1, CGs are produced by many different plants, some of which are either common household plants (e.g., *Nerium oleander*) [27,28] or can be mistaken for other herbs [29,30], thus, increasing the risk of accidental, or even purposeful CG poisoning. All the aforementioned CGs belong to the so-called cardenolides, which are, with few exceptions, produced exclusively by plants. Because of this, CGs have been primarily known as plant secondary metabolites, although information also exists on their production by animals (Table 2).

In the case of cardenolides, they are referred to as endogenous CGs, which are produced in humans by the adrenal cortex [36]. The second group of CGs comprises the so-called bufadienolides, which are also found in plants, for example, proscillaridin in the plant *Drimia maritima* [37]. However, bufadienolides are also present to a greater extent in animals, e.g., marinobufagenin in the toad *Rhinella marina* and humans [31,34] and bufotoxin in *Bufo bufo* gargarizans [35]. Another occurrence of bufadienolides is summarized in Steyn et al. [38]. It is also worth mentioning that the name of the whole group of bufadienolides is derived from the toads of the genus *Bufo*.

## 3. Production of Cardiac Glycosides

**Figure 4:**
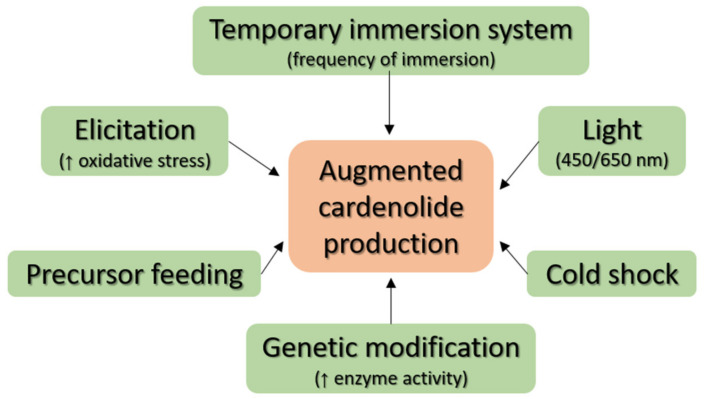
Production optimization of cardenolides in *Digitalis* plants.

Although the plants containing CGs have been known to mankind for several centuries, **Dgt** was not isolated from *Digitalis purpurea* until 1875, when it was achieved by German pharmacologist O. Schmiedeberg [39]. **Dg** had to wait another 55 years for its first isolation, which was achieved in 1930 by the English chemist S. Smith, who isolated it from *Digitalis lanata* [40]. Currently, **Dg** is known in the United States of America primarily under the tradenames Lanoxin and **Dgt** as Crystodigin. Nevertheless, for commercial purposes, both compounds are still obtained by isolation from the plants *Digitalis lanata* and *Digitalis purpurea*. Therefore, the aim is to optimize the cultivation process of these two plants as much as possible to achieve higher yields of **Dg** and **Dgt** as well as other medicinally relevant CGs (Figure 4). These optimizations can be done by several approaches, either by feeding the plants with CG precursors, production elicitation, different cultivation, or genetic engineering.

### 3.1. Precursor Feeding and Elicitation

**Figure 5:**
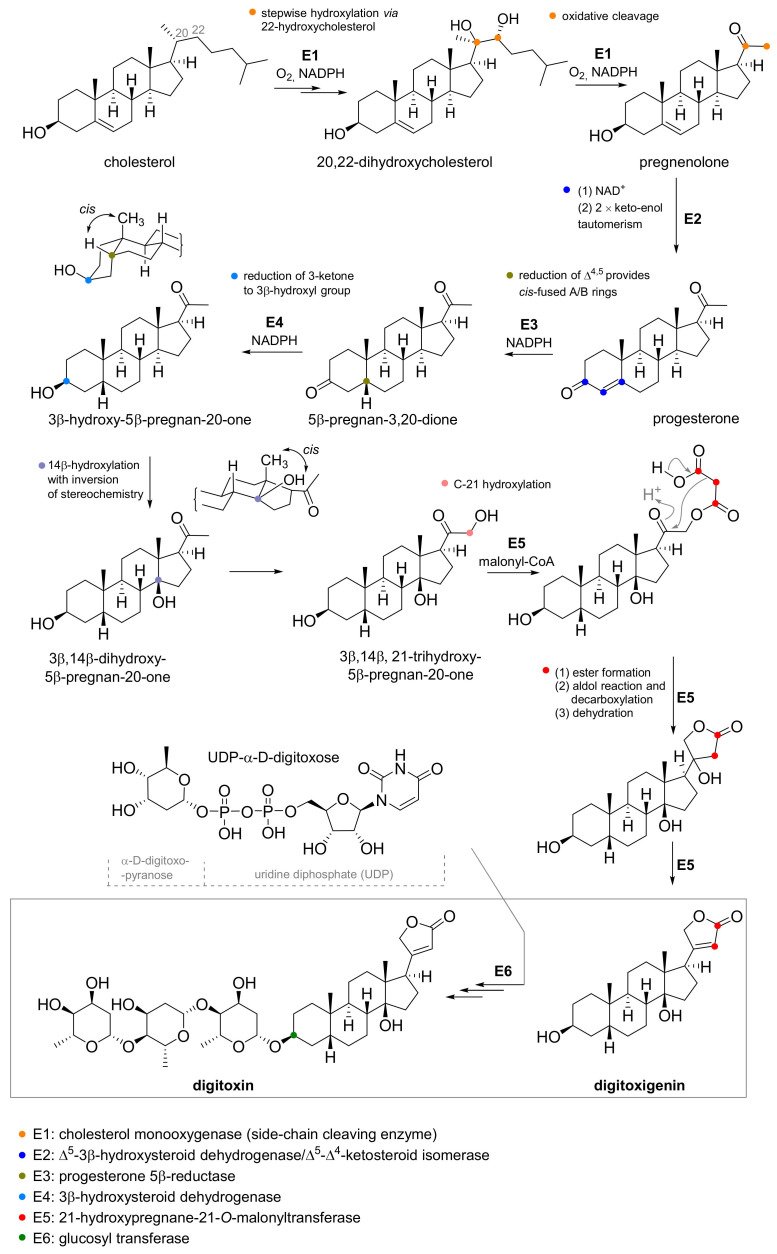
Biosynthesis of digitoxin in *Digitalis purpurea* and *Digitalis lanata* (differently colored dots highlight the structural change caused via enzymatic transformation).

CG production is affected by several factors, the first and probably also the most important one is the sufficient supply of necessary nutrients. Higher accumulation of CGs in *Digitalis lanata* and *Digitalis purpurea* was detected *in vitro* with the supply of both: suitable carbohydrates (sucrose, glucose, and raffinose) [41] and steroids, which in part serve as precursors for CG biosynthesis (Figure 5)—cholesterol, progesterone [42,43], 21-hydroxypregnane, 5β-pregnan-3,20-dione, 3β-hydroxy-5β-pregnan-20-one, 3β,14β,21-trihydroxy-5β-pregnane-20-one [44].

Furthermore, CG production can also be increased with SiO_2_-based elicitors (Silioplant^®^), chitosan oligosaccharide (Chitoplant^®^), and methyl jasmonate. All mentioned elicitors significantly increase the CG *in vitro* production in *Digitalis lanata* by elevating oxidative stress due to the increase in hydrogen peroxide and malondialdehyde (an indicator of lipid peroxidation) levels [45]. It is oxidative stress which is associated with increased CG production [46].

### 3.2. Cultivation Techniques

Another way to increase CG production is to cultivate *Digitalis purpurea* using biotechnology. In such a case, the plants are cultivated in a controlled environment, thus, eliminating the issues associated with nutrient supply, weather fluctuations, seasonal changes, and pests. One of the possible biotechnological production methods is the use of the so-called temporary immersion system, in which plants are exposed to the nutrient medium for a limited time at precisely defined intervals [47]. For the production of CGs in *Digitalis purpurea*, this method of cultivation was first described in 2009, when it was found that the production of biomass, as well as compounds 1 and 2, are affected by the frequency of immersions [48]. Another possibility is to cultivate undifferentiated *Digitalis purpurea* cells, as shown in Hagimory et al. [49]; however, these undifferentiated cells produce significantly less of **Dgt** compared to shoot-forming cultures, which are often used for temporary immersion system cultivation. For this reason, culturing undifferentiated cells from *Digitalis purpurea* for the mass production of CGs is not very promising.

### 3.3. Genetic Engineering

Another way to increase CG production is to use genetic engineering. In general, efforts to increase the production of secondary metabolites may target metabolic enzymes, regulatory elements, or other genes. Figure 5 shows the biosynthetic pathway of digitoxin in *Digitalis purpurea* and *Digitalis lanata*. Glycosylation steps from digitoxigenin to digitoxin use glycosyltransferases with UDP-α-d-digitoxose as a donor of digitoxose. This pathway offers opportunities for intervention by genetic engineering.

Gram-negative bacteria *Agrobacterium tumefaciens* and *Agrobacterium rhizogenes* are often used for genetic modification of plants, which can introduce their genetic information into the host plant using the so-called Ti or Ri plasmids, respectively [50,51]. This approach has been used in several studies for both *Digitalis purpurea* and *Digitalis lanata* [52,53,54,55,56]. This method is subsequently used for the identification of key enzymes involved in CG biosynthesis. One of these enzymes is progesterone-5β-reductase (EC 1.3.1.3). Kairuz et al. introduced the gene for this enzyme from *Arabidopsis thaliana* into *Digitalis purpurea* [57]. In this way, the prepared strain of *Digitalis purpurea*, the production of compounds **Dg** and **Dgt** increased two and four times, respectively.

Moreover, high throughput sequencing of RNA from *Digitalis purpurea* [58] is also used to predict sequences of enzymes involved in the cardiac glycoside biosynthesis pathway and series of non-coding RNAs with regulatory roles. These findings can then be used in a synthetic biology approach to address cardiac glycoside biosynthesis. Recently, genes coding selected enzymes from *Digitalis lanata*, *Arabidopsis thaliana,* bacterium *Comamonas testosteronii*, mouse (*Mus musculus*), and cattle (*Bos taurus*) were cloned into two vectors, which were inserted into yeast cells (*Saccharomyces cerevisiae*). These cells were then able to produce 3β,21-dihydroxy-5β-pregnan-20-one [59].

### 3.4. Physical Factors

Last, but not least, other factors that are being studied in connection with the increased accumulation of secondary metabolites in plants are physical factors such as the effect of light and temperature. It is known that plants, whether cultivated in the field or in laboratory conditions, need light to live. Verma et al. studied the effect of different wavelengths of light on the level of accumulation of **Dg** and **Dgt** in *Digitalis purpurea*, in which the combination of red (maximum intensity at 650 nm, 20%) and blue (maximum intensity at 450 nm, 80%) parts of the visible spectra have proved to be the most efficient [60]. As for the temperature, Wietmarschen et al. [11] cultivated *Digitalis lanata* at 15–25 °C with a cold shock, which significantly increased the production of **Dg** and, thus, it underlines the importance of stress factors for increased CG production.

## 4. Structure of Cardiac Glycosides

**Figure 6:**
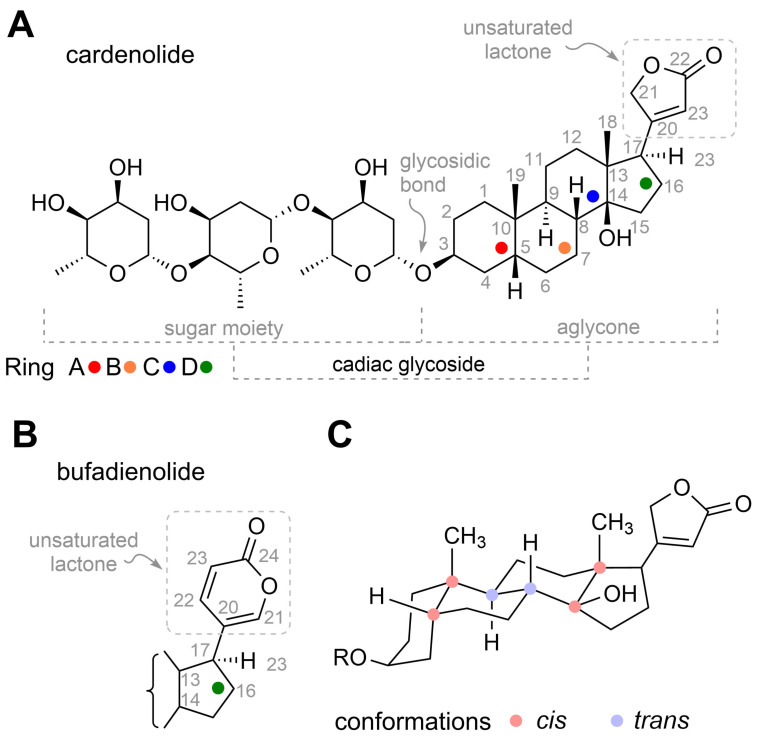
Chemical structure of digitoxin with highlighted parts of the molecule (**A**). Chemical structure of unsaturated lactone ring in bufadienolides (**B**). Ring conformation of a steroid skeleton (**C**).

The structure of CGs, like other steroid substances, is derived from the steroid skeleton (steran). The steroid skeleton generally consists of three six-membered carbon rings (A, B, C) and one five-membered ring (D) [Figure 6C]. Rings A and B are joined in a *cis* conformation, as are rings C and D. In contrast, rings B and C are joined in a *trans* conformation. The main substituents, typical of CGs, are then attached to this steroid skeleton, namely the unsaturated lactone ring C-17 in the β position and the saccharide in the C-3 position (Figure 6A). According to the type of lactone cycle, CGs are divided into the already mentioned cardenolides, which contain an unsaturated five-membered furanone ring (Figure 6A), and bufadienolides (Figure 6B), which contain an unsaturated six-membered pyranone ring. The attached carbohydrates are different, but most often they are d-digitoxopyranosyl, d-glucopyranosyl, d-oleandropyranosyl, l-rhamnopyranosyl, d-cymaropyranosyl, d-xylopyranosyl (Figure 2 and Figure 3). All CGs are further substituted at C-10 and C-13 at the β positions by a methyl group and C-14 also at the β position with a hydroxyl group. Substituents in other positions are individual for a given derivative. Common substitutions include those at C-12 and C-16 (Figure 2) or C-5 and C-10 (Figure 3). In most cases, these are also methyl or hydroxyl groups; however, in some cases, there is also a carbonyl group, an esterified hydroxyl group, or an epoxide. Sometimes a double bond also occurs in the structure of the steroid skeleton.

## 5. Na^+^/K^+^-ATPase Binding of Cardiac Glycosides

The most important molecular target of CGs is the already mentioned NKA, which is located in the cytoplasmic membrane of animal cells. The main task of NKA in cells is the transport of Na^+^ and K^+^ ions across the cytoplasmic membrane against a concentration gradient. To do this, NKA uses the energy released from the hydrolysis of adenosine triphosphate (ATP), which leads to conformational changes that affect the affinity of these ions to the protein and thus enable their transport. These conformational changes are the first factor that affects CG’s affinity for NKA. It was confirmed that NKA is in the so-called high-affinity state if it is phosphorylated and K^+^ binding has not yet taken place. In contrast, the change to a low-affinity state occurs after the binding of K^+^ ions, which are therefore agonists of CGs [61]. The affinity of CGs for NKA is also affected by another ion, Mg^2+^. As shown in a stability study of the ouabain-NKA complex, the complex is more stable at higher Mg^2+^ relative to K^+^ due to the competition between these two ions [62]. Besides, Mg^2+^ ions in NKA induce conformational changes that lead to its transition to a high-affinity state.

**Figure 7:**
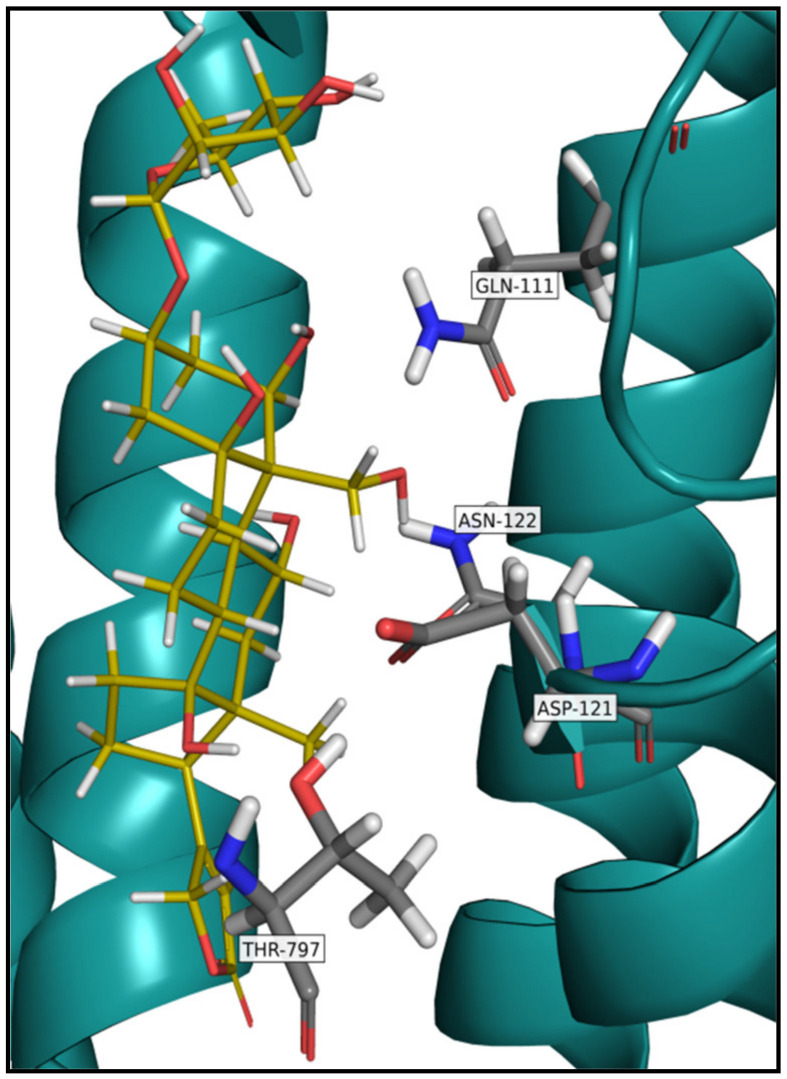
The crystal structure of Na^+^/K^+^-ATPase with bound ouabain. Highlighted are amino acid residues which in the case of mutation, have a significant impact on cardiac glycoside binding.

The second factor that affects the affinity of CGs for NKA and is closely related to the first is the amino acid composition of the binding site. The CGs binding site consists of six α-helices located in the transmembrane domain of the α-subunit of this protein and is accessible from the extracellular space [62]. This binding site can be divided into a non-polar portion composed of l-Ile_315_, l-Phe_316_, l-Gly_319_, l-Phe_783_, l-Phe_786,_ and l-Leu_793_ based on the polarity of the amino acids present and a polar portion composed of l-Gln_111_, l-Glu_117_, l-Asp_121_, l-Asn_122_, and l-Thr_797_ [62]. The amino acids l-Gln_111_, l-Asn_122,_ and l-Thr_797_, which form hydrogen bonds with the hydroxyl groups of the CG steroid skeleton, are particularly important for the binding of CGs to NKA. Substitution of these amino acids leads to a significant reduction in the NKA sensitivity to CGs, for example, IC_50_ of **Dg**, **Dgt,** and ouabain is increased by three orders of magnitude [63], and, therefore, is the reason for the resistance of some animal species such as mice, rats, and some insect species to CGs. Substitutions of l-Asp_121_ for l-Gly or l-Glu, l-Asn_122_ for l-His, or l-Asp, l-Gln_111_ for l-Thr or l-Arg, and l-Thr_797_ for l-Ala (Figure 7) were found in these organisms [7,64,65].

**Figure 8:**
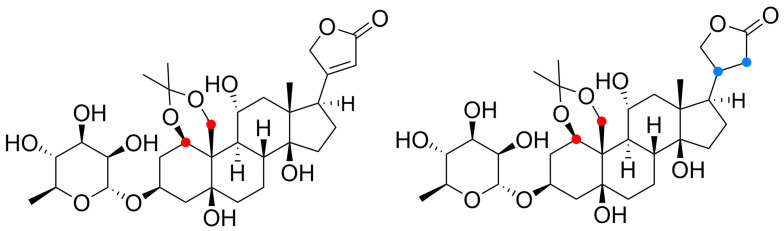
Chemical structures of acetonides derived from ouabain (differently colored dots highlight the structural change caused by chemical transformation).

Other substitutions have been also described. These, however, have less impact on NKA resistance to CGs than those described above. Namely, they are the following: l-Leu_330_ for l-Gln, l-Ala331 for l-Gly, l-Thr_338_ for l-Ala, l-Thr_338_ for l-Asn, and l-Phe_982_ for l-Ser [66]. The importance of polar interactions for CG binding to NKA is underlined by the work of Magpusao et al., who blocked the hydroxyl groups of ouabain at the C-1 and C19 positions with an acetonide group (Figure 8) [67]. In this case, the resulting derivative showed a 120-fold increase in half-maximal inhibitory concentration (IC_50_) compared to the parent compound.

**Figure 9:**
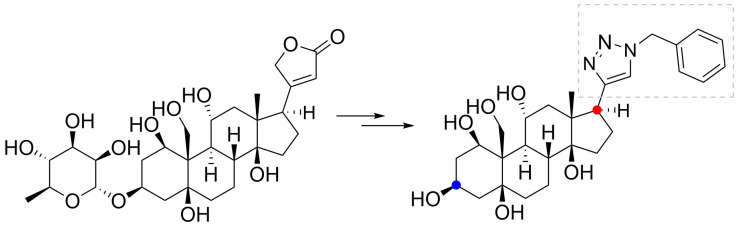
Triazole derived from ouabain (differently colored dots highlight the structural change caused by chemical transformation).

Another part of the CG molecules that significantly contributes to their binding to NKA is the lactone cycle, which in the case of cardenolides is five-membered and contains a double bond. It is its saturation or opening of the whole cycle that results in a significant reduction in the inhibitory activity of the resulting ouabain derivative [68]. The presence of a hydroxyl group at the C-21 position in **Dg** also leads to a decrease in the inhibitory activity of the resulting derivative due to inappropriate rotation of the steroid skeleton at the NKA binding site [69]. However, other derivatives of **Dg** were also prepared and exhibited higher inhibitory activity compared to **Dg** and mainly enhanced selectivity for some NKA isoforms, which are in the case of the α-subunit four of them. These cases involved particularly the introduction of a benzyl group at the C-21 position of **Dg**. The benzyl group was subsequently substituted by short non-oxygen and ether groups and depending on the type of the attached substituent, there were changes in compound selectivity (almost over 100% in case of non-oxygen derivative) for NKA isoforms α1, α2, α3 [70]. Syeda et al. went even further and decided to replace the entire furanone cycle of ouabain with a triazole, to which various hydrocarbon residues were attached at the N-1 position of the triazole (Figure 9). In the case of a bound benzyl residue, there was a reduction of the IC_50_ against the α4 NKA isoform by three orders of magnitude compared to the parent compound ouabain. On the contrary, in the case of the other isoforms, the derivatives of ouabain exhibited an increase in IC_50_ [71].

The last part of the CG molecule, which also plays an important role in their binding to NKA, and also affects the polarity of the whole molecule, is the sugar moiety. CG activity increases with decreasing degree of glycosylation; however, this rule is broken for aglycones, since aglycones generally exhibit lower inhibitory activity toward NKA than glycosylated forms [72], although exceptions exist. Petschenka et al. found that digoxigenin showed approximately three times higher IC_50_ on lamb NKA compared to **Dg [73]**. Importantly, not only the number of sugars moieties attached to the CG core but also the type of sugar significantly affects CG selectivity for the individual NKA isoforms. For example, oleandrin, which contains l-oleandrose in its molecule, was approximately three and two times more selective for the α1 and α3 NKA isoforms, respectively, compared to the α2 NKA isoform. In contrast, ouabain containing l-rhamnose exhibited no selectivity [74].

## 6. Biological Activity of the Most Important Cardiac Glycosides

The mechanism of action of **Dg** and **Dgt** and other CGs is the already mentioned inhibition of NKA, which disrupts the physiological concentration of Na^+^ and K^+^ ions. The Na^+^/Ca^2+^ exchanger then reacts to the augmented intracellular Na^+^ concentration by reversing the direction of transport, which results in an increase in the intracellular Ca^2+^ concentration. This in turn leads to enhanced muscle contraction [75]. However, the cell also responds to increased Ca^2+^ levels through other mechanisms. The endoplasmic reticulum (ER) serves the cell as the main Ca^2+^ reservoir. Active transport to the ER is ensured by sarco-/endoplasmic reticular Ca^2+^-ATPase [76,77], which contributes to the maintenance of the cytosolic Ca^2+^ concentration at a relatively low level (ca. 100 nM) [78]. However, in the case of a significant and especially a long-term increase in the cytosolic concentration of Ca^2+^, this mechanism ceases to be sufficient. In such a case, other organelles must be involved in the regulation of Ca^2+^, namely mitochondria, which in part also serve as a reservoir (or “buffer”) of Ca^2+^. Nevertheless, an excessive increase in Ca^2+^ concentration in the mitochondria disrupts the electrochemical potential of the mitochondrial membrane, which begins to rupture gradually, releasing cytochrome c into the cytosol and activating the apoptotic pathway [79,80,81]. Moreover, inhibition of NKA also leads to depletion of intracellular K^+^ which is also one of the hallmarks of apoptosis, because it leads to cell shrinkage, activation of caspases, and DNA fragmentation [82,83,84]. Disruption of ion homeostasis is considered to be one of the most important mechanisms in relation to the anticancer activity of CGs.

**Figure 10:**
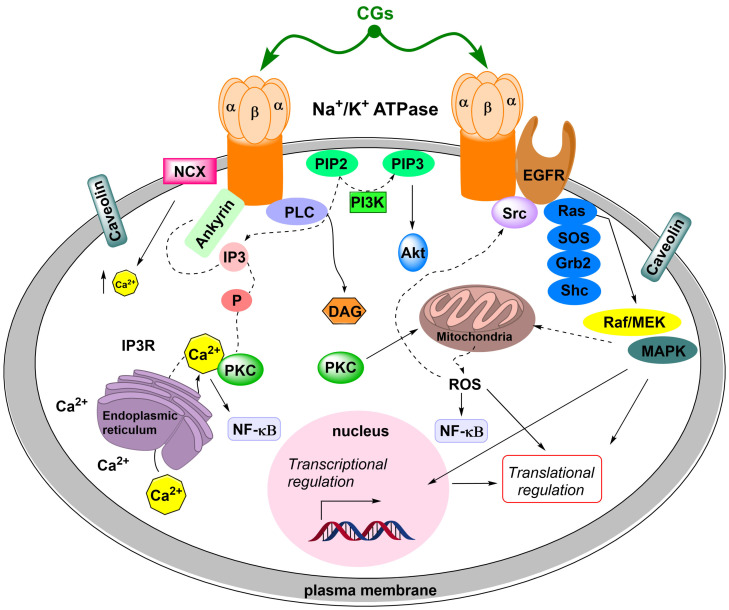
A signaling complex of Na^+^/K^+^-ATPase is composed of multiple structural proteins (ankyrin—involved in organizing the Na^+^/K^+^-ATPase: IP_3_R complex and caveolin), receptors (IP_3_R—inositol 1,4,5-trisphosphate receptor, EGFR—epidermal growth factor receptor), and protein and lipid kinases (Src-kinase, PI3K). CGs binding to Na^+^/K^+^-ATPase induce conformational changes in the enzyme which affect its interactions with other intracellular proteins. CGs binding activates the Src/EGFR/Ras/Raf/MEK/ERK kinase (ERK—extracellular-signal-regulated protein kinase) cascade and the PI3K/Akt (PI3K—phosphatidylinositol 3-kinase, Akt—protein kinase B) pathway. These events promote PLC-catalyzed (PLC; phospholipase C) production of IP_3_ (inositol 1,4,5-trisphosphate) and DAG (diacylglycerol), which activate IP_3_R in the endoplasmic reticulum membrane and PKC (protein kinase C). IP_3_R is the Ca^2+^ channel that releases Ca^2+^ (yellow octagon) from the endoplasmic reticulum to the cytoplasm in response to an IP_3_ increase. NCX—Na^+^/Ca2+ exchanger; PIP_2_—phosphatidylinositol 4,5-bisphosphate; PIP_3_—phosphatidylinositol 3,4,5-trisphosphate; ROS—reactive oxygen species.

The second mechanism of action of **Dg** and **Dgt**, and other CGs, is also associated with NKA, but in this case, NKA lacks its transport function and, conversely, exhibits a signaling function. In this case, NKA forms a signaling complex in the caveolae of the cytoplasmic membrane together with caveolin, non-receptor tyrosine kinase (SrcK), epidermal growth factor receptor (EGFR), phospholipase C and phosphatidylinositol-3-kinase [85,86,87], see Figure 10 for summarization. In this situation, three signaling cascades are activated after the interaction of CGs with NKA:

(i) SrcK/EGFR—in this case, activated SrcK transactivates EGFR, which in turn activates the Ras/Raf/MEK/MAPK pathway [88,89,90]. This activates transcription factors and generates reactive oxygen species. Reactive oxygen species subsequently interact with the NKA signalosome, which activates other SrcK molecules and, thus, amplifies the SrcK/EGFR pathway signal [91,92].
(ii) SrcK/phospholipase C—activated phospholipase C hydrolyzes the ester bond of phosphatidylinositol-4,5-bisphosphate and the released inositol-1,4,5-triphosphate subsequently interacts with inositol triphosphate receptors on the ER, the opening of which causes Ca^2+^ oscillation [93,94]. Ca^2+^ oscillations subsequently induce the activation of the antiapoptotic subunit p65 of the nuclear factor kappa-light-chain-enhancer of activated B cells (NF-κB), which serves as a transcription factor and increases the production of the antiapoptotic factor Bcl-xL from the Bcl-2 family of proteins [95].
(iii) The latter signaling pathway is associated with phosphatidylinositol-3-kinase. This signaling is not dependent on SrcK [96] and, conversely, involves the activation of phosphoinositide-dependent protein kinase-1 (PDK-1). Activated PDK-1 subsequently activates protein kinases B and C [97,98].

Effects on other cellular targets have also been described for CGs, most of which are dependent on NKA signaling. These include inhibition of focal adhesion kinase and related inhibition of new vascular bundle growth (angiogenesis), inhibition of hypoxia-induced factor 1α (HIF-1α) production, increase of p21^Cip1^ cell cycle inhibitor production, inhibition of topoisomerase I and II, inhibition of steroid receptor coactivators 1 and 3 (SRC-1 and 3), and the opening of volume-regulated anion channels. All of these effects are related to the anticancer activity of CGs, however, their antiviral effects have recently been discovered. A particular CG effect has been described on human immunodeficiency virus (HIV), hepatitis C virus, human papillomavirus, and cytomegalovirus (HCMV). The following chapters summarize the roles of CGs in the most important indications.

### 6.1. Heart Disease and Blood Pressure

If we omit the use of CG in the period before W. Withering, the first therapeutic use of CGs was the treatment of heart failure and cardiac arrhythmias. The use of CGs for this indication is based on their well-researched action as NKA inhibitors, thereby, disrupting the ionic homeostasis of the cell. As already mentioned in Chapter 5, inhibition of NKA by CGs subsequently causes an increased intracellular Ca^2+^ concentration, thereby, enhancing cardiac muscle contraction. This condition is called the positive inotropic effect [75]. Currently, only **Dg** is used in most countries to treat these heart diseases, although both derivatives, **Dg** and **Dgt**, act by the same mechanism. However, in Norway, for example, **Dgt** is preferred since it is eliminated from the body by non-urinary routes to a higher extent compared to **Dg**. As it is shown by **a** mathematical model which fits observed data [99] for **Dgt** and **Dg**, the rate of their elimination is 61.6 and 41%, respectively. Therefore, the excretion of **Dgt** is less dependent on renal function. This fact makes it a more suitable therapeutic, especially for elderly patients who may suffer from impaired kidney function. On the other hand, **Dgt** shows a longer elimination half-life (~8 days) compared to **Dg** (~36 h), which is thus more rapidly eliminated from the body upon intoxication (the observed mean of therapeutic serum concentrations of **Dg** are ca. 1.4 ng·mL^−1^, toxic concentrations are 2–3 times higher) [100], and for this reason, it is generally more preferred for medicinal use [101,102].

Apart from the role of CGs in the therapy of heart diseases, their action is also related to increased blood pressure. In the past, patients with hypertension have been found to have elevated levels of so-called endogenous ouabain, a substance structurally identical to plant ouabain [103]. Moreover, two isomers of endogenous ouabain that differ in polarity were discovered quite recently [104]. Endogenous ouabain is a substance that is produced in the human body by the adrenal cortex [36] and along with other endogenous CGs plays a role in the regulation of blood pressure. This role has been confirmed by several studies [105,106,107,108,109]. Agunanne et al. investigated the effect of commercial Digibind^®^ against marinobufagenin, another endogenous CGs, on blood pressure in a pregnant rat model. Digibind^®^, which consists of antigen-binding fragments commonly used in **Dg** intoxication, was able to significantly reduce rat blood pressure (by 25%) after its administration (10 mg·kg^−1^ every day from day 10 to 20 of pregnancy; animals were continuously injected by deoxycorticosterone acetate and their drinking water was replaced with 0.9% saline) by binding marinobufagenin in plasma [106]. It has also been confirmed that the plasma levels of endogenous ouabain increase in response to central nervous system stimulation by angiotensin II (2.5 ng·min^−1^ for 14 days), again leading to higher blood pressure [104]. Another way, in which endogenous CGs could contribute to higher blood pressure is the effect on vascular vasoconstriction, which is caused by NKA inhibition and a consequent increase in intracellular Ca^2+^ levels. The mechanism of this increase of blood pressure, which is summarized in Blaustein et al. [107]) is probably also related to the fact that endogenous ouabain is synthesized in the body in response to higher sodium excretion (twice the rate of a baseline excretion) [105]. The last mechanism of blood pressure increase is related to the NKA/SrcK signalosome when after activation of the Ras/Raf/MEK/MAPK signaling cascade, cardiac hypertrophy occurs (infusion of 15 µg·kg^−1^ per day of ouabain into rats for 18 weeks), which is associated with higher pumping capabilities of the heart and subsequently leads to increased blood pressure [108].

### 6.2. Cardiac Glycosides and Cancer

The reason why are CGs nowadays so extensively studied is due to their promising anticancer potential. The cytotoxic effect of CGs was first reported in 1967 [109]. Shiratori evaluated the activity of 37 compounds isolated mainly from *Digitalis purpurea* in HeLa-S3 cancer cells although subsequent *in vivo* experiments in mice did not show any anticancer activity of the CGs (probably caused by resistant NKA isoform present in mice). However, already in that time, the authors speculated that the cytotoxic effect of CGs is probably associated with a membrane protein exhibiting ATPase activity. After more than 10 years, Stenkvist et al. [110] published a short study on the morphology of breast cancer cells, which were smaller and less prone to metastasis in patients taking CGs. This study was subsequently supported by another one showing that patients on CG therapy have a 9.6 times lower risk of cancer recurrence five years after mastectomy [111].

The cytotoxic action of CGs in a wide range of cancer cell lines is currently known; however, the exact mechanism of action has not been reliably elucidated, yet. Nevertheless, it is clear that in most cases, the cytotoxic action of CGs involves interaction with NKA. The first discovered anticancer mechanism of CG action was their ability to induce apoptosis in cancer cells *in vitro*, which was controlled by changes in the ionic balance after the NKA inhibition by CGs [79,80,81]. To disrupt the ionic homeostasis of cells *in vitro*, a relatively high concentration of CGs is needed, it is commonly in tens to hundreds of nanomolar concentrations depending on the given CG derivative [72].

Other anticancer mechanisms of CGs are also associated with NKA; however, in this case, the concentration of CGs must be significantly lower, in low nanomolar concentrations, units of nM maximum. In this case, the NKA signaling cascade described in Chapter 6 is triggered. Although these steps are often associated with the induction of cell proliferation, the opposite effects have been reported for some cancer cell lines, i.e., inhibition of cell proliferation and induction of apoptosis [112,113,114,115,116,117,118,119,120,121,122]. This is based on the fact that this signaling pathway is connected with other pathways associated with proliferation inhibition and cytotoxic effects. Activation of these pathways is likely to predominate in cancer cells, and for this reason, CGs have different effects on cancerous and noncancerous cell lines.

Several of these anticancer mechanisms were described in the past. The first is the inhibition of focal adhesion kinase, which subsequently suppresses motility of human cells from lung carcinoma (A549) and canine kidney cells (MSV-MDCK) *in vitro* and, conversely, promotes cell adhesion, leading to a reduction in the metastatic potential of these cells and also to a reduced ability to form new vascular bundles (angiogenesis) [112,113]. Another anticancer mechanism of CGs is cell cycle arrest caused by increasing the production of the cell cycle inhibitor p21^Cip1^ in estrogen receptor-negative human breast cancer cells (MDA-MB-435s) [114]. p21^Cip1^ was previously shown to arrest the cell cycle in the G1 and G2 phases *in vitro* by inhibiting various cyclin-dependent kinases [115,116,117]. Besides suppressing cell motility and arresting the cell cycle, inhibition of HIF-1α production has been also linked to NKA signaling. HIF-1α regulates the expression of proangiogenic genes, specifically vascular endothelial growth factor and the angiopoietin/Tie-2 system *in vitro* in primary human endothelial cells [118], thereby, improving tumor blood flow. Consequently, inhibition of HIF-1α production results in reduced angiogenesis, thereby, reducing tumor development which was observed *in vitro* in human hepatoblastoma cells transfected with reporter genes for hypoxia-inducible expression of firefly luciferase (Hep3B-c1), CD133 positive human glioma stem cells (GSC), and human glioma cells (U-87MG) [119,120,121].

Furthermore, the presence of volume-regulated anion channels (VRAC) has been demonstrated in membrane microdomains along with NKA signalosome. Crosstalk between NKA and VRAC is likely to be mediated by nicotinamide adenine dinucleotide phosphate oxidase and reactive oxygen species. Activated VRAC then reduces cell volume and induces anticancer activity. This mechanism was observed *in vitro* only in colorectal cancer cells (HT-29) and, conversely, was not observed in noncancerous primary human fibroblasts (Hs68) or human embryonic cells (HEK 293T) [123].

Besides NKA signaling, another molecular target of CGs was found. In 2006, it was proven that CGs inhibit the activity of topoisomerase I and II, the stress-reducing enzymes participating in the overwinding or underwinding of DNA [124]. This happens independently of NKA signaling. Interestingly, from three evaluated CGs (proscillaridin A, **Dg**, and ouabain), only proscillaridin A can inhibit topoisomerase I activity in breast cancer cells (MCF-7) at 100nM concentration, compared to **Dg** and ouabain, which did not exhibit inhibitory activity even at 100 µM concentration. In contrast, all these three CGs inhibited topoisomerase II activity indicating that the pyranone cycle of bufadienolides or changes in the distribution of substituents on the steroid CG skeleton is probably responsible for the specific inhibition of topoisomerase I [125]. Moreover, CGs, namely Dg and bufalin, inhibit the transcriptional activity of SRC-1 and SRC-3. These receptors are growth regulators belonging to the p160 family of nuclear receptor coactivators and are often overexpressed in human breast cancer positive on the epidermal growth factor receptor 2 [126,127,128] or recurring prostate cancer [129,130]. Wang et al. showed that Dg and bufalin inhibited both SRC-1 and SRC-3 and, thus, blocked *in vitro* proliferation of HeLa cells transfected with reporter genes [131]. Another anticancer mechanism of CGs, also independent of NKA, is altering the membrane fluidity. This phenomenon was observed for oleandrin in human histiocytic lymphoma cells [132]. The alterations in the plasma membrane fluidity in HL-60 cells differentiated into neutrophils led to down-regulation of interleukin-8 receptors which are commonly up-regulated in many tumors [133]. These three mechanisms underline the fact that NKA is not the only target of CGs and that maybe in the future more targets of CGs will be discovered.

### 6.3. Antiviral Activity of Cardiac Glycosides

Apart from potential cancer treatment, the antiviral activity of **Dg** and **Dgt** against HIV was recently discovered [134]. By activating the NKA signalosome, **Dg** and **Dgt** can inhibit the expression of the viral genome. This is independent of changes in intracellular Ca^2+^ concentration, indicating that CGs suppress HIV protein expression of HIV envelope proteins (Env) and group-specific antigen (Gag) polyprotein at concentrations at which NKA is not inhibited [135]. In particular, **Dg** was found to reduce the activity of serine/arginine-rich protein kinases (SRPK) from the cell division control protein 2-like kinases (CLK) family involved in RNA splicing, thereby affecting alternative RNA splicing and suppressing virus replication *in vitro* in human primary cells obtained from healthy or HIV-infected individuals [136].

Researchers also discovered the antiviral activity of CGs against HCMV, which involves two different mechanisms. The first one is associated with the production of ether-à-go-go related gene family K^+^ channels (ErgCh) and NF-κB. Both proteins are overproduced in HCMV-affected cells and, conversely, after culturing with **Dg**, **Dgt,** and ouabain at nanomolar concentrations, both ErgCh and NF-κB production decreased, which correlated with the ability of CGs to suppress HCMV immediate-early, early, and late protein production [137]. The second mechanism is related to adenosine monophosphate-activated protein kinase (AMPK) activation and autophagy. This activation is associated with the interaction of CGs with the α1 subunit of NKA. In human foreskin fibroblasts (HFF) infected by HCMV, unc-51 like autophagy activating kinase 1 (ULK1) is phosphorylated by the mammalian target of rapamycin (mTOR) on l-Ser_757_, resulting in suppression of autophagy. In contrast, in **Dgt**-treated cells, ULK1 is phosphorylated on l-Ser_317_ and, conversely, mTOR activity is suppressed, inducing autophagy, and inhibiting HCMV [138] Both mechanisms seems to be connected with NKA signaling activity.

Besides HIV and HCMV, the antiviral properties of CGs against other viruses, such as herpes simplex virus type 1 (HSV-1), influenza virus, or Middle East respiratory syndrome coronavirus (MERS-CoV) were found. In the case of HSV-1, viral gene expression and virus release are probably inhibited by changes in intracellular Na^+^ concentration [139], because as reported by Zhang et al. [140], HSV-1 replication in primary neuronal cultures *in vitro* significantly increased (5–8 times) after blocking Na^+^ channels by tetrodotoxin. Another virus, the replication of which can be inhibited by ouabain, is the influenza virus. The viral inhibition is mediated by a decrease in the intracellular concentration of K^+^, caused by *in vitro* NKA inhibition by ouabain in lung carcinoma cells (A549). A similar effect was observed after cultivating these cells in a medium depleted for K^+^. Both cases led to inhibition of protein translation and were independent of NKA signaling [141]. In the last-mentioned virus, MERS-CoV, CG antiviral activity (unlike in the influenza virus) is associated with NKA signaling, as a blockade of virus entry into the cell has been reported at low CG concentrations (50 nM of ouabain or 10–15 nM of bufalin) *in vitro* in hepatocellular carcinoma (Huh-7) cells [142].

## 7. Conclusions

CGs as plant toxins have been known to mankind for centuries and during that time, they have built up a solid place in pharmacy as drugs for the treatment of heart failure and arrhythmias due to their positive inotropic effect on the heart muscle cells. Besides, they have been shown to be cytotoxic or to suppress cancer cell proliferation [143] by interacting with NKA by mechanisms that differ from their action on healthy cells. The first part of this review article focuses on the occurrence of CGs and the optimization of their production, which is still a major challenge, as CGs are currently still isolated from plants, unlike many other drugs that are produced by organic synthesis or microbial biotechnology. The next part was devoted to the CG structure concerning their ability to interact with NKA. This research is connected with the study of CGs as anticancer drugs [144,145]. The therapeutic window of these compounds is very narrow, which complicates their use in clinical practice not only in the field of cardiology but also as potential anticancer drugs. Although the anticancer mechanism of CGs is based mainly on activation of the NKA signalosome which occurs at their non-toxic concentrations, efforts have been made to further reduce the systemic toxicity of CGs by employing sophisticated drug delivery strategies to target them into tumors. One approach is to use peptide-based targeting, which was used in the case of periplocymarin, a cardiac glycoside isolated from *Periploca* plants. This compound was conjugated with octreotide, a cyclic octapeptide mimicking somatostatin hormone. This conjugation widened the therapeutic window *in vitro* on various cancer cell lines and also increased the anticancer effect *in vivo* [146]. Another approach to widen the therapeutic window is to deliver CGs using nanoparticles based on chitosan and a synthetic polymer [147]. The antiviral activity of CGs described in the last part also employs a various range of different mechanisms dependent on a specific viral infection. Some of them are caused by ion disbalance driven by NKA inhibition (HSV-1, influenza virus), others, as in the case of anticancer activity, by activation of NKA signalosome (HIV, MERS-CoV, HCMV). Summarized, CGs have come a long way over the centuries from arrow poisons to life-saving drugs. Besides, new findings suggest that their journey may not be nearing the end.

## Figures and Tables

**Table 1:** An overview of the most important producers of cardiac glycosides.

Source	Cardiac Glycoside	Reference
*Strophanthus kombe* (Apocynaceae)	K-strophanthoside, cymarin, helveticoside, strophanthidin, erysimoside, k-strophanthin-β, neoglucoerysimoside	[12,13]
*Strophanthus gratus* (Apocynaceae)	G-strophanthin (ouabain)	[14]
*Digitalis lanata* (Scrophulariaceae)	Digoxigenin, deacetyllanatoside C, digoxigenin-bis-digitoxoside, gitoxigenin, digoxin, lanatoside C, digitoxigenin, α-acetyldigoxin, β-acetyldigoxin, lanatoside B, gitoxin, lanatoside A, digitoxin	[15]
*Digitalis purpurea* (Scrophulariaceae)	Digitoxin, digitoxigenin, gitoxin, gitoxigenin, gitaloxin, glucodigitoxin, glucogitoxin, glucogitaloxin	[16,17,18]
*Nerium oleander* (Apocynaceae)	Oleandrin, neritaloside, cardenolide B-1, oleagenin, odoroside H, oleaside A, neriaside	[19,20]
*Coronilla varia* (Fabaceae)	Hyrcanoside, deglucohyrcanoside	[21]
*Convallaria majalis* (Liliaceae)	Convallatoxin, perconval, canariengenin, rhodexin, periplorhamnoside, convallatoxol, peripalloside, strophalloside, strophanolloside, deglucocheirotoxin, lukondjoside, convalloside, deglucocheirotoxol, periguloside, rhodexoside	[22,23,24,25]
*Apocynum cannabinum* (Apocynaceae)	Strophanthidin, cymarin, cynocannoside, helveticoside, apobioside, apocannoside, cannogenol	[26]

**Table 2:** Examples of cardiac glycosides produced by animals.

Source	Cardiac Glycoside	Reference
*Homo sapiens sapiens*	Ouabain (endogenous), marinobufagenin	[31]
*Canis lupus familiaris*	Ouabain (endogenous)	[32]
Wistar rats	Ouabain (endogenous)	[33]
*Rhinella marina*	Marinobufagenin	[34]
*Bufo bufo* gargarizans	Bufotoxin	[35]

## Data Availability

Not applicable.

## Abbreviations

AMPK	Adenosine monophosphate-activated protein kinase
ATP	Adenosine triphosphate
CG	Cardiac glycoside
CLK	Cell division control protein 2-like kinases
Dg	Digoxin
Dgt	Digitoxin
DNA	Deoxyribonucleic acid
EGFR	Epidermal growth factor receptor
Env	Envelope proteins
ER	Endoplasmic reticulum
ErgCh	Ether-à-go-go related gene family K^+^ channels
Gag	Group-specific antigen
Hs68	Primary human fibroblasts
HCMV	Human cytomegalovirus
HEK 293T	Human embryonic cells
HeLa-S3	Human cervix carcinoma cells (subclone)
HIF-1α	Hypoxia-induced factor 1α
HIV	Human immunodeficiency virus
HSV-1	Herpes simplex virus type 1
HT-29	Human cells from colorectal carcinoma
IC_50_	Half-maximal inhibitory concentration
MAPK	Mitogen-activated protein kinase
MEK	Mitogen-activated protein kinase kinase
MERS-CoV	Middle East respiratory syndrome coronavirus
mTOR	Mammalian target of rapamycin
NF-κB	Nuclear factor kappa-light-chain-enhancer of activated B cells
NKA	Na^+^/K^+^-ATPase
PDK-1	Phosphoinositide-dependent protein kinase-1
Raf	Serine/threonine kinase
Ras	Rat sarcoma protein
RNA	Ribonucleic acid
siRNA	Short interfering ribonucleic acid
SRPK	Serine/arginine-rich protein kinases
SrcK	Non-receptor tyrosine kinase
SRC-1	Steroid receptor coactivator 1
SRC-3	Steroid receptor coactivator 3
ULK1	Unc-51 like autophagy activating kinase 1
VRAC	Volume-regulated anion channels
